# Cancer Neuroscience: Linking Neuronal Plasticity with Brain Tumor Growth and Resistance

**DOI:** 10.3390/biology15020108

**Published:** 2026-01-06

**Authors:** Doaa S. R. Khafaga, Youssef Basem, Hager Mohamed AlAtar, Abanoub Sherif, Alamer Ata, Fayek Sabry, Manar T. El-Morsy, Shimaa S. Attia

**Affiliations:** 1 Department of Basic Medical Sciences, Health Sector, Galala University, New Galala City 43511, Suez, Egypt; 2 Medical and Pharmaceutical Industrial Biotechnology Department, College of Biotechnology, Misr University for Science and Technology (MUST), 6th of October City 12566, Giza, Egypt; 200025117@must.edu.eg (Y.B.); 200033381@must.edu.eg (A.S.); 200025070@must.edu.eg (A.A.); 200023335@must.edu.eg (F.S.); 3 Zoology & Entomology Department, Science College, Al-Azhar University, Naser City 11884, Egypt; HajarAlAttar104461@azhar.edu.eg; 4 Faculty of Nanotechnology for Postgraduate Studies, Cairo University, Giza 12613, Egypt; 14222023442600@pg.cu.edu.eg; 5 Anatomy & Embryology Department, Faculty of Medicine, Ain-Shams University, Abaseya 11566, Cairo, Egypt; 6 Biomedical Sciences Department, College of Medicine, Gulf Medical University, Ajman 4184, United Arab Emirates

**Keywords:** cancer neuroscience, neuronal plasticity, glioblastoma, neuroglioma synapses, tumor microenvironment

## Abstract

Brain tumors, particularly glioblastoma, remain among the most lethal cancers, with limited survival benefits from current genetic and molecular-targeted approaches. Emerging evidence reveals that beyond oncogenes and mutations, neuronal plasticity, long-term potentiation, synaptic remodeling, and neurotransmitter-driven signaling play a pivotal role in shaping tumor progression and therapeutic response. This convergence of neuroscience and oncology has given rise to the field of cancer neuroscience, which explores the bidirectional interactions between neurons and malignant cells. In this review, we summarize fundamental principles of neuronal plasticity, contrasting physiological roles with pathological reprogramming in brain tumors. We highlight how tumor cells exploit synaptic input, particularly glutamatergic signaling, to enhance proliferation, invasion, and integration into neural circuits. We further discuss how neuronal-driven feedback loops contribute to therapy resistance, including chemoresistance, radioresistance, and immune evasion, mediated through pathways such as mitogen-activated protein kinase (MAPK), phosphoinositide 3-kinase/protein kinase B (PI3K/AKT), and calcium influx. The tumor microenvironment, including astrocytes, microglia, and oligodendrocyte-lineage cells, emerges as an active participant in reinforcing this neuron-tumor ecosystem. Finally, this review explores therapeutic opportunities targeting neuronal plasticity, spanning pharmacological interventions, neuromodulation approaches (transcranial magnetic stimulation (TMS), deep brain stimulation (DBS), optogenetics), and computational/artificial intelligence frameworks that model neuron tumor networks to predict personalized therapy. Also, we propose future directions integrating connect omics, neuroinformatics, and brain organoid models to refine translational strategies.

## Simple Summary

Brain tumors, particularly glioblastoma, are among the most aggressive and treatment-resistant malignancies of the central nervous system. While traditional research has focused primarily on genetic mutations and oncogenic signaling pathways, recent discoveries demonstrate that neuronal activity and neuronal plasticity actively contribute to tumor growth, invasion, and resistance to therapy. This review highlights the emerging field of cancer neuroscience, focusing on how pathological reprogramming of neuronal plasticity supports brain tumor progression. In addition, we discuss key mechanisms linking neuronal signaling to oncogenic pathways, including MAPK and PI3K/AKT, as well as the contributions of astrocytes, microglia, and other components of the tumor microenvironment. By highlighting future directions involving connect-omics and brain organoid platforms, this review aims to provide a conceptual framework for translating cancer neuroscience insights into novel, mechanism-based therapies for brain tumors.

## 1. Introduction

Primary tumors of the brain and central nervous system (CNS) constitute a critical global health burden, with GLOBOCAN data reporting over 300,000 new diagnoses and nearly 250,000 related deaths each year [1]. The findings from the Global Burden of Disease (GBD) project further highlight rising incidence and mortality rates, particularly in low- and middle-income regions, reflecting widening global inequities [2]. Glioblastoma (GBM) remains the most prevalent and lethal form of primary brain cancer, responsible for the majority of CNS tumor–associated deaths [3]. Even with maximal therapy surgical resection, radiotherapy, and temozolomide chemotherapy median survival rarely exceeds 15 months, and the 5-year survival rate remains below 10% [4]. Historically, GBM research has focused on genetic and molecular aberrations such as Isocitrate Dehydrogenase (IDH) mutations, EGFR amplification, and TERT promoter alterations, which underpin the 2021 World Health Organization (WHO) classification system. While these advances have improved diagnostic and prognostic stratification, they have delivered only marginal therapeutic benefits [5]. Extensive clinical and translational investigations demonstrate that interventions directed at single molecular pathways have been insufficient against GBM’s profound adaptability and heterogeneity [6]. Resistance mechanisms frequently circumvent such targeted approaches, curtailing their long-term efficacy [7].

This therapeutic impasse has shifted perspectives, emphasizing that the nervous system is not merely a passive environment but an active participant in tumor progression [8]. The interdisciplinary field of cancer neuroscience has consequently emerged, integrating oncology, neurobiology, and immunology to explore the reciprocal interactions between neurons and cancer cells [9]. Compelling evidence now shows that neuronal activity directly enhances glioma growth. For example, optogenetic stimulation of neuronal firing accelerates tumor proliferation [10], and neuroligin-3, a synaptic protein released during neuronal activity, functions as a powerful glioma mitogen [11]. Recent Studies have identified genuine neuron–glioma synapses, whereby glutamatergic signaling through AMPA receptors depolarizes glioma cells, driving both invasion and proliferation [12]. Such synaptic integration allows tumors to exploit neuronal plasticity and embed themselves within neural networks. At the molecular level, signaling cascades linked to neuronal plasticity including PI3K/AKT, MAPK, and calcium-dependent pathways promote malignant behaviors and therapy resistance [13]. AMPA receptor–driven activity has also been implicated in enhanced glioma invasiveness and perivascular migration, linking synaptic remodeling with tumor dissemination [13]. Furthermore, glioma-associated neuronal plasticity undermines treatment efficacy by enabling adaptive rewiring following chemotherapy or radiotherapy, maintaining stem-like tumor subpopulations, and altering drug sensitivity profiles. Neurotransmitter imbalances, such as elevated glutamate and reduced GABAergic signaling, additionally modulate therapeutic responsiveness [12]. The tumor microenvironment, including astrocytes, microglia, and oligodendrocyte lineage cells, further sustains this neuron–tumor ecosystem by providing metabolic, trophic, and inflammatory support [14]. This review article seeks to investigate the current understanding of how neuronal plasticity across synaptic, structural, and functional dimensions fuels brain tumor progression and therapeutic resistance. By integrating insights from neuroscience, oncology, and computational biology, we aim to lay the groundwork for advancing precision neuro-oncology [15].

## 2. Methodology

**Figure 1:**
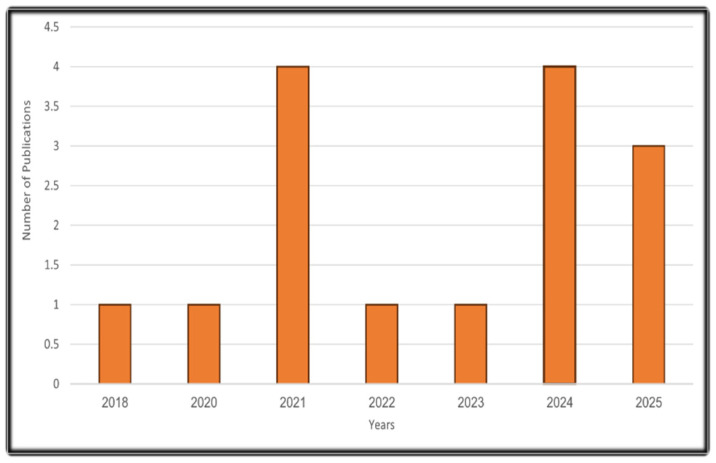
Year-wise distribution of the 167 publications included in this review based on the PubMed database (2018–2025) following application of the predefined inclusion and exclusion criteria.

We conducted a structured narrative review of peer-reviewed literature from 2018–2025 focused on neuronal plasticity and neuron–glioma coupling in brain-tumor progression and therapy. Searches were based on PubMed, Scopus, Web of Science, ScienceDirect, and Google Scholar using keywords; (glioblastoma OR glioma OR brain tumors) AND (neuronal OR synaptic neuronal plasticity OR LTP OR synaptic remodeling OR “neuroglioma synapse”) AND (glutamatergic OR AMPA OR NMDA OR “calcium influx” OR MAPK OR PI3K/AKT) AND (astrocytes OR microglia OR oligodendrocyte OR “tumor microenvironment” OR resistance OR neuromodulation OR (TMS OR DBS OR optogenetics) OR organoids OR connect omics OR “single-cell” OR (computational OR AI)). We included experimental studies, and high-quality reviews/case reports that directly examined synaptic input to cancer cells, neuron–tumor interactions, or neuronal-linked resistance. We excluded non-English articles, conference abstracts without full texts, and studies lacking a brain-tumor or neuronal-plasticity context, as illustrated in Figure 1.

## 3. Fundamentals of Neural Plasticity

**Figure 2:**
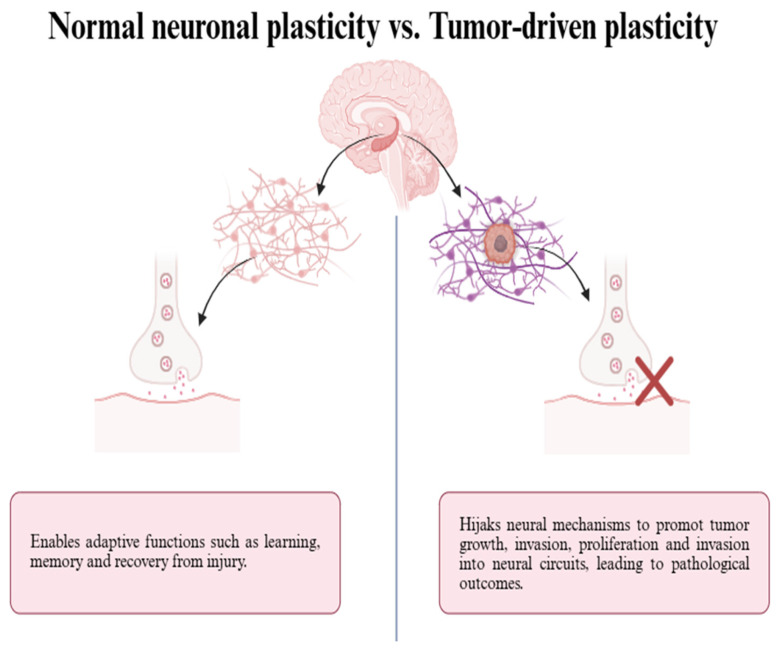
Comparative schematic illustrating differences between Normal (physiological) neural plasticity and Tumor-driven plasticity. In the healthy brain, neural plasticity is driven by activity-dependent synaptic signaling, balanced excitation-inhibition, and experience-mediated circuit refinement. In tumor-driven plasticity, glioma cells exploit physiological neural plasticity mechanisms such as synaptogenesis, neurotransmitter signaling, and activity-dependent gene regulation to reshape local neural networks in favor of malignancy. Created by Biorender, (Biorender.com, accessed on 3 December 2025).

Neural plasticity is the ability of the nervous system to change both physically and functionally in response to experience or injury stimuli. Both adults and children exhibit this ability during development. Different levels of the nervous system’s organization exhibit neuronal plasticity. So, neural plasticity will be divided into neuronal or glial neuronal plasticity, synaptic neuronal plasticity, nervous tissue plasticity (e.g., hippocampus, prefrontal cortex, amygdala, and so on), etc. [16]. The peculiar form of neurons and their capacity for chemical and electrical communication through complex synaptic connections make them distinctive both structurally and practically. In particular, synaptic neuronal plasticity describes the structural and functional changes in synapses that affect the potency and effectiveness of neuronal communication [17]. As illustrated in Figure 2, while normal neuronal plasticity supports learning, memory formation, and tissue repair without disturbing neurotransmitter transmission, tumor-driven neuronal plasticity induces aberrant remodeling of neural circuits, leading to pathological outcomes. Long-term depression (LTD) and hippocampal long-term potentiation (LTP) are Hebbian types of synaptic neuronal plasticity that are commonly thought to represent the physiological correlates of associative learning. The dynamic interaction between LTD and LTP promotes the development of intricate associative memories that are difficult to generalize [18]. While LTD, which lowers synaptic weights, has frequently been relegated to the category of “counterpart to LTP,” which prevents synaptic saturation, LTP, which raises synaptic weights, has historically been given a key role in learning and memory [19]. Long-term potentiation in neuronal plasticity refers to the fortification of synapses after repeated synaptic stimulation.

As N-methyl-D-aspartate receptor (NMDAR) activity is inhibited by a magnesium block, basal firing of synapses primarily recruits α-amino-3-hydroxy-5-methyl-4-isoxazolepropionic acid receptor (AMPARs). High-frequency stimulation of presynaptic neurons increases the electrical efficiency of the synapse. By recruiting the NMDARs to let the inflow of calcium ions, sodium influx through AMPAR opening sufficiently depolarizes the postsynaptic terminal to remove the magnesium barrier [20]. Calcium is a secondary messenger that can enhance postsynaptic responsiveness by causing alterations that contribute to LTP development [20]. The postsynaptic response that results will therefore be greater than its prestimulation level. LTP is thought to be the neural correlate of memory and learning. Additionally, in animal models, the administration of pharmacological drugs that disrupt LTP led to learning impairment or memory retention failure. When combined, LTP is regarded as a well-known cellular model for learning and memory [21]. For the neural system, damping and attenuating impulses are also essential. How we can relearn a new concept or give up a contradictory one is not something that LTP alone addresses. According to behavioural theory, animals must adjust to ongoing environmental changes that could conflict with previously acquired knowledge. Therefore, the synaptic framework of declining synaptic strengths is LTD [20]. Due to technical difficulties, LTD was previously a difficult phenomenon to notice since it could be mistaken for the result of technical errors. Nonetheless, its observation has been consistently documented, and progress has been made to clarify its chemical mechanisms and memory function. Two forms of LTD have been thoroughly investigated: one is mostly produced by NMDAR-dependent synaptic activation (NMDAR-LTD), while the other is induced through mGluR activation (mGluR-LTD). When it comes to flexibility during learning, LTD plays a part. This is because retraining is necessary to “update” old memories [22]. A remarkable mechanism known as brain structural neuronal plasticity enables the mature brain to learn, adapt to changes in the environment, heal from injuries or illnesses, and delay the ageing process [23]. In neural networks and, consequently, the cortex, information processing and storage are determined by the connection between neurons, or the number of synapses and their transmission efficiency weights. Therefore, we must comprehend how cortical neural networks create their connectivity to comprehend how they function. The first mechanism, known as structural or architectural neuronal plasticity, creates and destroys synapses between neurones. In general, two main processes can alter connection. The second phase, known as synaptic neuronal plasticity, alters the transmission efficiency of these synapses. Donald O. Hebb first suggested synaptic plasticity. Subsequent research revealed that neural activity causes the synaptic transmission efficacy to continuously enhance and diminish. Different pre- and postsynaptic action potential timings are significant in addition to firing frequencies [24]. It has also been demonstrated that, on a longer time scale, synaptic weights can be homoeostatically controlled to achieve a specific network firing frequency. On the one hand, structural neuronal plasticity describes the retraction and extension of axons and dendrites, which mostly occurs during developmental stages or following significant damage to the network structure. Conversely, it relates to the most common process in adult networks, the formation and removal of synapses. Since most cortical synapses are located on what are known as dendritic spines, we may study their dynamics to obtain insight into synaptic dynamics. Because they are extremely mobile, spines can appear and disappear over the course of hours or days. It has been demonstrated that their head-volume affects how long they live [25]. Additionally, the strength of the excitatory postsynaptic potentials (EPSPs) from the associated synapse the electrophysiological counterpart of synaptic weight correlates with this head-volume. Additionally, it has been demonstrated that stimulation regimens that increase or decrease synaptic weight cause the spine head to grow or contract, respectively. Therefore, the spine head volume, which dictates the likelihood of structural alterations, is influenced by synaptic neuronal plasticity. This suggests that synaptic and structural neuronal plasticity interact strongly [24]. Structural neuronal plasticity includes dendritic spine density and shape, as well as the size of the neural arbour, including dendritic length and number of ramifications. Dendritic spines are tiny projections that accept excitatory inputs and function as postsynaptic biochemical compartments. Based on their shape, they are divided into five classes: cup-shaped, stubby, mushroom, thin, and filopodium. Their formation and elimination are dependent on the activity [26]. These protrusions are mostly present in glutamatergic neurones, including Purkinje cells in the cerebellum, CA3 neurones in the hippocampus, pyramidal neurones in the neocortex, medium spiny neurones in the striatum, and a tiny population of GABAergic interneurons, which are primarily found in the hippocampus. Glutamate-induced activation of NMDA and AMPA receptors aids in the maturation of dendritic spines [27]. Furthermore, γ-aminobutyric acid (GABA) inhibits local Ca^2+^ signaling to regulate the competitive selection of dendritic spines. Actin and microtubule dynamics govern structural neuronal plasticity. Since they are required to establish the morphology of the spine, they are both important regulators of the dendritic spine [26], where structural neuronal plasticity involves axon growth and repair mechanisms, dendritic spine creation, and neurogenesis promotion [28]. After a variety of events, many medicines can promote axonal development. An axon that a disease process has severed may stretch from the severed end, which would be the most basic growth response. The formation of a collateral from the proximal section of a damaged axon is another example of growth. On the other hand, fibre systems that are next to or parallel to a cut pathway but are not themselves injured can also promote axonal growth [29]. The development from either cut or undamaged fibres over moderate distances is referred to as sprouting [29]. Axonal sprouting, the development of new dendritic spines, and synaptic connections are examples of experience-dependent morphological alterations that make up structural neuronal plasticity [30]. Injured axons must reseal damaged terminals, rebuild the cytoskeleton, produce and transport building blocks, put together axon components, and form growth cones to successfully regenerate [31]. At more localized levels, anatomical alterations may also be seen, such as rearranging dendritic spines and axonal varicosities or changing the shape of branches in terminal fields. Such alterations may have significant functional significance despite being more physically restricted. It is well recognized that many types of neuronal plasticity entail biochemical modifications to synaptic efficacy without requiring morphological rearrangement [29]. Different features of circuit dynamics may be regulated by both intrinsic and synaptic neuronal plasticity, which can be activated separately or concurrently. Whether synaptic neuronal plasticity and Hyperpolarization-activated Current/Ih (IHP) are triggered by the same activity sensors is yet unknown because the activity perturbations employed to investigate homeostatic neuronal plasticity concurrently disrupt several calcium-dependent signaling pathways. It has been determined that there are numerous additional types of compensatory neuronal plasticity that may support network stability [32]. Heterosynaptic neuronal plasticity meta-neuronal plasticity, and presynaptic homeostatic plasticity are among them. Voltage-gated ion channels are regulated by activity, just like ligand-gated ion channels that mediate synaptic transmissions, and intrinsic neuronal plasticity has been shown to play a role in certain types of learning and memory. Naturally, neurones control their intrinsic excitability by negative feedback as well. Homeostatic regulation of intrinsic excitability, or IHP, is a common mechanism in the central nervous system and plays crucial roles in circuit homeostasis [33]. This was first shown in invertebrate central pattern generator neurones, where outward and inward conductance are actively adjusted to maintain burst firing. Subsequent research in neocortical and hippocampus neurones confirmed this. In addition to the quantity, intensity, and timing of synaptic inputs, the neuron’s input-output function, that is, the amount of current input needed to cause the neuron to fire output, determines the neuron’s excitability. It is determined by the distribution of ionic conductance throughout the entire neuron as well as the characteristics of the passive wire. The identity and membrane distribution of voltage-gated ion channels are key determinants of intrinsic excitability. In order to homeostatically control excitability, neurones need to be able to recognize changes from a certain activity threshold and initiate signal transduction cascades that control the distribution and abundance of ion channels. As the reader may see, this necessitates the involvement of several molecular players, cell biological processes, and signaling channels [32]. Increased excitability may be caused by changes in sub- and suprathreshold membrane conductances that trigger an action potential. Recent studies demonstrated that changes in input resistance as the mechanism cause increases in excitability in terms of passive characteristics. Our findings are consistent with several studies that have demonstrated changes in input resistance as the mechanism causing increases in excitability in terms of passive characteristics. The suppression of G-protein-coupled inwardly rectifying K^+^ channels (GIRK channels) or cAMP-responsive element-binding protein (CREB)-dependent control of excitability by decreasing K^+^ conductance or potassium and sodium voltage-gated conductances could be the cause of the increase in excitability there. H-channels’ role in intrinsic membrane characteristics is also mentioned. The suppression of GIRK channels or CREB-dependent excitability control by decreasing K^+^ conductance or potassium and sodium voltage-gated conductances could be the cause of the increase in excitability there. H-channels’ role in intrinsic membrane characteristics is also mentioned [34]. The comprehensive connection between the neuronal and neurotransmission systems is crucial for identifying the extraordinary adaptability of human brain function, despite dependence on established anatomical connectivity. The neurotransmitter system employs a distinct set of differential equations that characterize the dynamics of neurotransmitter concentration, as defined by the determined Michaelis–Menten release-and-reuptake dynamics [35]. In a study, they used a variety of positive excitatory and negative inhibitory inputs to stimulate the model, which changed the neuronal masses’ mean firing rate and mean membrane potential. This type of stimulation was selected because it was thought to be comparable to common paradigms of electrical or sensory transcranial direct current stimulation (tDCS) [36]. In the instance of glutamate (Glu), we discovered that there was a general movement of NT from the vesicles to the cytosol under excitatory stimulation. This was explained by an enhanced firing rate for the excitatory population. An elevated Glu MRS signal in response to excitatory stimulation would represent this movement of Glu from the imperceptible vesicular compartment to the more visible Extracellular space (ECS) and cytosolic compartments [36]. Numerous experimental studies that have demonstrated elevated Glu after excitatory sensory stimulation are consistent with this. Regarding GABA, recent studies demonstrate that the vesicular pool increased in tandem with a decrease in NT in the ECS and cytosolic pools. In reaction to an excitation, this would manifest as a reduced GABA MRS signal [36]. It has been proposed that anodal stimulation causes a decrease in the MRS GABA signal because of decreased activity of glutamic acid decarboxylase (GAD)-67, an enzyme involved in GABA synthesis that decreases with increased excitatory firing. Excitatory input to the model can be thought of as analogous to sensory stimulation or anodal tDCS. While these processes are beyond the scope of this work, we note that the model predictions are in agreement with the actual results regardless of their absence [36]. Neuronal plasticity is the capacity of cells to undergo reprogramming, alter their identity and destiny, and restore homeostasis and tissue regeneration after injury. Additionally, pathological circumstances like cancer are facilitated by cell neuronal plasticity, which allows cells to transition between different cell states and acquire new phenotypic and functional characteristics that aid in tumour development, progression, metastasis, and treatment resistance [37].

## 4. Neuronal Plasticity Driving Brain Tumor Growth

### 4.1. Neuroglioma Synapses

The progression of high-grade gliomas (HGG) is influenced not only by tumor-intrinsic factors but also by the surrounding neural microenvironment. One key mechanism involves the release of neuroligin-3 (NLGN3) from both neurons and precursor glial cells, driven by the activity of ADAM10. NLGN3 activates the PI3K–mTOR signaling pathway, which, in turn, promotes synapse-related transcriptional programs, driving glioma growth. Xenograft models further demonstrate that gliomas fail to progress without NLGN3, and pharmacological inhibition of ADAM10 successfully impedes its release, thus slowing tumor growth. This highlights NLGN3 as a central driver of neuron-tumor interactions and positions it as a promising therapeutic target for HGG [38].

Moreover, recent studies on diffuse midline gliomas (DMGs) have revealed that neuronal activity accelerates tumor progression via mechanisms beyond glutamatergic signaling. Specifically, glioma cells in DMGs are subjected to excitatory GABAergic inputs due to abnormal chloride regulation mediated by NKCC1. This depolarization promotes tumor proliferation, with lorazepam treatment exacerbating this effect and leading to faster tumor growth and reduced survival in animal models. In contrast, hemispheric HGGs show a weak response to GABAergic inputs, indicating a subtype-specific interaction. These findings suggest that GABAergic signaling is a crucial factor in DMG progression, offering potential new avenues for therapeutic intervention [39].

### 4.2. Excitatory Glutamatergic Signaling

HGGs, which include DMGs and hemispheric glioblastomas, are highly aggressive, invasive, and heterogeneous tumors. Recent studies have highlighted the complexity of interactions between gliomas and their microenvironment, unveiling distinct mechanisms that drive tumor progression and resistance. In DMGs, GABAergic synapses rather than glutamatergic ones play a key role in tumor growth. These synapses, activated by GABAA receptors, paradoxically cause depolarization in glioma cells due to irregular chloride regulation by NKCC1 transporters. This depolarization promotes tumor proliferation, and the use of lorazepam, which enhances GABAergic signaling, accelerates tumor growth in xenograft models. On the other hand, hemispheric HGGs show little GABAergic depolarization, suggesting that GABAergic signaling is particularly crucial for DMG progression. This difference opens potential avenues for subtype-specific treatments targeting GABAergic signaling in DMGs [40].

In addition to neuronal activity, recent findings highlight the role of tumor microtubules (TMs) in glioma resistance and recurrence. TM-positive glioma cells exhibit stem-like properties, express neural stem cell markers, and share characteristics with immature neurons and cancer stem cells. These TMs support invasion, resistance, and recurrence, indicating that they may be a key feature driving glioma malignancy. The overlap between TM-positive cells and cancer stem cells suggests that targeting these structures may provide new therapeutic strategies for resistant glioma subpopulations [41].

Further complicating the progression of gliomas, the tumor microenvironment is characterized by distinct niches that influence glioma cell adaptability and resistance. Two critical niches in glioblastoma are the perivascular niche (PVN) and the multicellular networks formed by TMs. Cells in the PVN are typically quiescent and exhibit an extraordinary ability to repair tumor damage post-therapy, relying heavily on active Notch Receptor 1 (NOTCH1) signaling for their maintenance. On the other hand, suppression of NOTCH1 promotes the extension of TMs, creating a cellular network that supports glioma cell survival and resistance to therapy. Notably, these two niches are interdependent, with strong cross-compensation between them, indicating that targeting only one niche may not be sufficient to overcome resistance. Understanding the interaction between these niches and their role in glioma progression highlights NOTCH1 as a critical molecular switch in the glioma microenvironment [42].

### 4.3. Neuron–Tumor Feedback Loops: Hyperexcitability Networks Fueling Tumor Expansion

Low-grade neuroepithelial tumors (LGNTs), particularly those exhibiting glioneuronal characteristics, are strongly associated with pharmacoresistant epilepsy. Despite extensive research, effective treatments for both seizures and tumor progression remain elusive. Recent advancements in animal models, particularly through intraventricular in utero electroporation (IUE), have allowed for the creation of LGNT-like brain tumors in mice. These models successfully replicate key genetic, molecular, and histological features of human LGNTs. The slow proliferation of LGNTs significantly contributes to their epileptogenic properties, highlighting the importance of studying the interactions between tumor cells and surrounding neuronal circuits. This emerging understanding of tumor–neuron crosstalk is critical for addressing the challenges of seizure development in these tumors [41].

Similarly, glioblastomas, which grow within cortical tissue, cause significant disruptions to neural network activity. These tumors induce persistent neural hyperexcitability, leading to cognitive deficits and seizures. Imaging studies in mouse models have shown that tumor invasion alters both neural activity and glutamate signaling, with abnormal glutamate accumulation extending beyond the areas typically governed by calcium signaling. The degree of network disruption varies depending on the tumor’s progression stage and proximity to surrounding cells. This information provides valuable insights for precision diagnostics and potential treatments aimed at mitigating the impact of glioblastoma on brain function [43].

Furthermore, seizures associated with gliomas are driven by a combination of excessive glutamate release and impaired inhibitory signaling. Research has demonstrated that in glioma-bearing mice, the peritumoral region experiences a loss of inhibitory interneurons and disrupted chloride homeostasis due to reduced expression of the Potassium-Chloride Cotransporter 2 (KCC2) co transporter. This alteration shifts GABAergic signaling from inhibitory to excitatory, which contributes to neuronal hyperexcitability and promotes seizure activity. While glutamate release alone does not fully explain the seizures, the combination of excessive glutamate and GABAergic disinhibition strongly amplifies the effects. These findings position KCC2 as a promising target for therapies aimed at preventing or treating glioma-associated epilepsy [44].

### 4.4. Tumor-Induced Remodeling of Brain Circuits

Glioblastomas, a form of high-grade glioma, significantly disrupt brain circuitry, negatively affecting neural function and contributing to shorter survival times in patients. These tumors alter normal brain networks by establishing new synaptic connections that not only support their own growth but also impair cognitive functions. Glioblastoma cells in highly connected brain regions secrete thrombospondin-1 (TSP1), a protein that promotes synapse formation and facilitates tumor-neuron communication. This disruption in neural circuits leads to worsened survival outcomes and cognitive impairments, including reduced language performance. Targeting TSP1 with drugs such as gabapentin has shown potential in limiting tumor proliferation, offering a new direction for treatment [43].

In addition to cognitive impairments, glioblastoma also alters cortical network dynamics at multiple spatial and temporal levels. The tumor’s growth within cortical circuits leads to persistent hyperexcitability, which can manifest as cognitive decline and seizures. Using calcium and glutamate imaging in mouse models, studies have demonstrated that as glioblastomas infiltrate brain tissue, neural activity becomes progressively dysregulated. Abnormal glutamate accumulation extends beyond areas of normal calcium signaling, suggesting a decoupling of excitatory processes. The extent of these disruptions varies depending on the tumor’s growth stage and proximity to local synaptic areas, underscoring the importance of precision diagnostics and tailored treatments for glioblastoma [45].

Furthermore, glioma-driven remodeling of neuronal circuits can induce regional immunosuppression, particularly in areas where glioblastoma cells integrate more strongly with neuronal circuits. TSP1 not only drives synapse formation and neuronal over activity but also dampens immune responses, primarily by recruiting anti-inflammatory tumor-associated macrophages. Blocking TSP1 or glutamatergic signaling can shift the tumor microenvironment toward a more pro-inflammatory state, encourage CD8+ T-cell infiltration, and improve survival in animal models. These findings suggest that targeting the interaction between tumor cells and the immune system could enhance the effectiveness of immunotherapies [45].

In diffuse gliomas, which are particularly invasive and resistant to treatment, tumors mimic developmental and neuronal programs to establish interconnected networks. These networks are formed through gap junctions linking tumor cells and astrocytes, as well as through direct synaptic and paracrine signaling with neurons. Such multicellular networks support glioma progression and survival, and understanding the molecular mechanisms behind these tumor–tumor and tumor–neuron–astrocyte interactions could lead to innovative therapeutic strategies aimed at disrupting these pathological brain networks [46].

## 5. Neuronal Plasticity and Therapy Resistance

### 5.1. Synaptic Rewiring as an Adaptive Mechanism After Radiotherapy and Chemotherapy

Neuron tumor synaptic interactions have now been defined as a significant biological axis in which gliomas actively hijack host circuitry: these interactions transform the tumor microenvironment into an electrophysiological niche that promotes survival, invasion, and regrowth [47]. High-resolution imaging has anatomical and functional detail, showing that malignant glioma cells can form bona-fide excitatory synapses with peri-tumoral neurons, creating direct neuron→glioma electrical coupling, leading to membrane depolarization producing proliferation and calcium signaling in tumour cells [48]. Human translational data demonstrate that increased tumor–network functional connectivity is associated with more aggressive clinical behavior and worse survival in patients, implying that synaptic integration is not merely noise but carries measurable selective advantage in patients [49]. Post-ionizing radiation or cytotoxic chemotherapy, the remnants of brain tissue are active biologically: treatment results in glial activation, cytokine and growth-factor release, blood–brain barrier and extracellular matrix remodelling, immune changes, and the combined processes create a permissive environment for mutant neuron–tumor interactions, including possible structural re-wiring [50]. These activity-dependent, neuronal-derived secreted proteins, most notably NLGN3, are paracrine mitogens that couple neuronal firing with transcriptional programs responsible for tumours, and activate pathways which control growth, synaptogenesis, and adaptive neuronal plasticity in glioma cells. Neurotrophic signaling through BDNF-TrkB axis further stabilizes and prolongs pathological synaptic stabilization. BDNF release increases synaptic efficacy, and TrkB activation in tumor cells further enhancing synaptic stabilization, producing downstream MAPK/PI3K signaling and malignancy neuronal plasticity [51]. Following genotoxic stress, some surviving glioma cells exhibit a collective behaviors indicative of a “plastic” state. neuronal plasticity reflects phenotypic changes that include cytoskeletal restructuring and upregulation of neurogenic gene programs, and increased membrane permeabilization to neuronal processes, and metabolic restructuring all of which help to provide repopulation potential [50]. Single-cell and spatial transcriptomics consistently reveal cellular subpopulations of tumor cells that preferentially occupy neuron-rich niches and demonstrate upregulated gene modules for neuronal synaptic proteins, neurotransmitter receptors and synaptic integration signatures that could support a model of niche specific selection [52]. Electrophysiological testing and in vivo imaging in particular show that treated tumour beds tend to develop a higher level of synchronous firing and develop epileptiform activity after treatment. This pathological hyperexcitability creates a positive feedback loop in which neuronal firing continues driving glioma growth and recruitment into the network [45]. Extracellular glutamate is a key chemical mediator of peritumoral hyperexcitability: tumor-induced upregulation of the cystine/glutamate antiporter xCT (SLC7A11) along with impaired astrocytic glutamate exposure results in the accumulation of glutamate which is harmful to adjacent neurons and increases excitatory signaling onto tumor cells [53]. In gliomas, dysregulated SLC7A11/xCT expression has a mechanistic link to redox adaptation, invasiveness, and therapy-resistance phenotypes, providing a molecular bridge from altered neurotransmitter handling to decreased sensitivity to chemo- and radiotherapy [54]. Ionotropic glutamate receptors expressed on tumour cells particularly Ca^2+^-permeable AMPA receptors (AMPARs) and NMDA receptors mediate rapid Na^+^/Ca^2+^ influxes that activate calcium-dependent kinases and immediate early transcriptional programs; perturbation of these receptors alters chemo- and radiosensitivity in preclinical models [55]. Beyond classical synapses, a multicompartmental physical infrastructure tumour microtubes, extracellular vesicle exchange, and remodeled synaptic scaffold proteins stabilizes neuron–tumour connections and permits long-range electrochemical and metabolic coupling across the malignant network [56]. Epigenomic and transcriptomic reprogramming accompanying neuronal engagement produces a neural-like malignant signature: chromatin remodeling and activity-dependent transcription sustain stemness, DNA-repair programs, and survival pathways that underlie durable adaptive resistance [57]. Crucially, multiple preclinical interventions that target different nodes of the rewiring cascade blocking activity-regulated mitogens (e.g., NLGN3 inhibitors), inhibiting AMPAR function (pharmacologic AMPAR antagonists), modulating glutamate export/clearance (xCT inhibitors or EAAT enhancers), or antagonizing TrkB signaling—have produced proof-of-principle reductions in tumour proliferation and re-sensitization to cytotoxic therapies [58].These converging data motivate a translational paradigm in which cytotoxic regimens are combined with circuit-directed therapies: rational combinations must be informed by patient-level biomarkers of tumour–neuron connectivity (functional connectivity mapping, neurotransmitter/metabolite signatures, synaptic-gene expression) to select individuals most likely to benefit while minimizing neurotoxicity [59]. In sum, synaptic rewiring following radiotherapy and chemotherapy is a multi-layered, active adaptive process—driven by activity-dependent secreted mitogens, neurotrophic support, neurotransmitter imbalance, structural network remodeling, and epigenetic reprogramming that selects for clones capable of exploiting host circuitry to survive, repopulate, and resist therapy [47].

### 5.2. Support of Glioma Stem-like Cells by Plasticity Changes & Neurotransmitter Imbalance

Glioma stem-like cells (GSCs) preferentially reside in neuron-rich perivascular and subcortical niches where ongoing neuronal activity supplies both synaptic inputs and activity-regulated soluble factors that reinforce self-renewal programs [48]. The exposure of GSCs to neuron-derived ligands, particularly NLGN3, stimulates rapid transcriptional reprogramming that activates proliferative and synaptogenic gene modules, effectively linking the spikes of neuronal activity to glioma clonogenic expansion [38]. BDNF released from active neurons engages TrkB receptors on GSCs and downstream effectors (MAPK/ERK and PI3K/AKT), thereby stabilizing nascent neuron–tumor contacts and promoting survival pathways that blunt the cytotoxic impact of chemoradiation [47]. In human studies, it has been reported that blocking TrkB and/or NLGN3 signalling in preclinical models decreases GSCs’ self-renewal ability and sensitizes tumours to DNA-damaging treatments, suggesting that activity-regulated trophic signals play a causal role in conferring therapy resistance [60]. Neuronal firing also alters the metabolic microenvironment: activity-dependent release of glutamate and other metabolites supports a metabolic coupling in which GSCs shift toward pathways that favor antioxidant defense and nucleotide synthesis needed for repair after genotoxic stress. A key mechanistic node is glutamate homeostasis: many gliomas overexpress the cystine/glutamate antiporter (SLC7A11/xCT), exporting glutamate into the extracellular space while importing cystine to fuel glutathione synthesis a change that simultaneously fosters neuronal hyperexcitability and protects tumor cells from oxidative damage induced by therapy [61]. Elevated extracellular glutamate creates a feed-forward loop: it enhances excitatory drive onto both neurons and tumor cells, sustains AMPAR/NMDAR signaling in GSCs, and promotes calcium-dependent survival signaling that reduces apoptosis after chemotherapy or irradiation [62]. Genomic and epigenomic profiling of resistant tumors reveal co-selection for SLC7A11 upregulation alongside transcriptional programs for DNA repair and ferroptosis resistance, linking altered neurotransmitter handling to concrete mechanisms of treatment escape [63]. Beyond glutamate, the immunometabolic consequences of neurotransmitter dysregulation matter: glutamate-rich microenvironments modulate local immune cells and can foster immunosuppressive niches that further permit GSC persistence after therapy [53]. GABA can exert both inhibitory and excitatory effects depending on the cellular context. While GABA signaling is traditionally inhibitory in mature neurons, in certain glioma subtypes, particularly diffuse midline gliomas, GABAergic inputs can be depolarizing due to altered chloride homeostasis maintained by NKCC1. Depolarizing GABAergic signaling has been shown to promote tumor cell proliferation and increase tolerance to therapy [64]. Experimental models using patient-derived xenografts and optogenetics demonstrate that both excitatory and aberrant inhibitory synaptic inputs can expand the GSC pool in vivo, indicating that synaptic modality is less important than the net effect of membrane depolarization and downstream calcium signaling [65]. Single-cell data further indicate that neuronal engagement induces a proneural-to-mesenchymal transition in subsets of GSCs, a phenotypic conversion associated with enhanced motility, invasiveness, and therapeutic resistance [66]. Calcium influx through AMPARs and NMDARs in GSCs activates CaMKs and calcineurin, which cooperate with MAPK and PI3K signaling to upregulate immediate-early genes and DNA-repair factors, thereby shortening repair latency and lowering the efficacy of DNA-targeting agents [67]. Metabolic rewiring accompanies these signaling changes: GSCs increase flux through glutamine and serine–glycine pathways to supply nucleotides and maintain redox balance, adaptations that are reinforced by neuron-derived substrates and that have been linked to chemoresistance [68]. From a therapeutic standpoint, inhibition of xCT to lower extracellular glutamate, pharmacologic AMPAR antagonists (to diminish excitatory synaptic drive), TrkB inhibitors (to block BDNF signaling), and NKCC1 blockers to reinstate inhibitory GABA responses reduce GSC frequency and restore sensitivity to conventional treatments to some degree in model systems [69]. For clinical translation, combining cytotoxic therapy with agents that normalize neurotransmitter balance or interrupt activity-regulated trophic signaling offers a rational path forward. Still, it requires careful biomarker-guided patient selection to avoid central neurotoxicity [69].

### 5.3. Molecular Cascades

**Figure 3:**
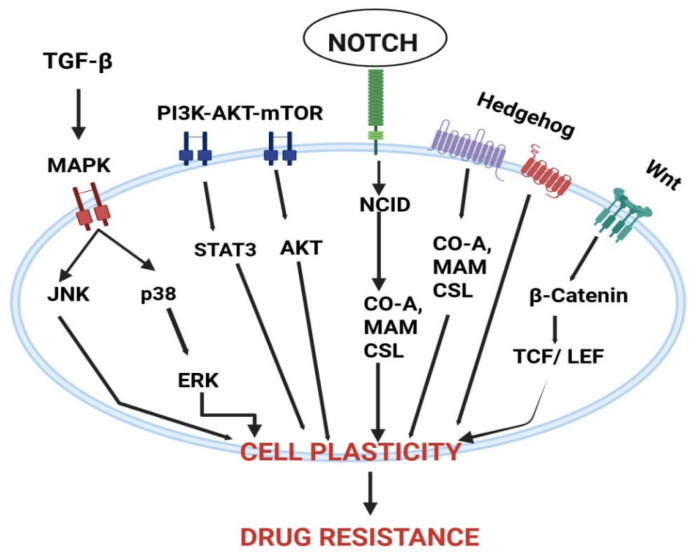
A schematic representation of pathway convergence from the NOTCH, MAPK, PI3K/AKT/mTOR, Wnt, and Hedgehog pathways, together with Ca^2+^ influx induced by neuron→tumor interactions, resulting in transcriptional/epigenetic reprogramming, metabolic adaptation (e.g., SLC7A11/xCT), and multicellular networks that promote therapeutic resistance. Created by Biorender, (Biorender.com, accessed on 3 December 2025).

Neuronal inputs convert transient electrical activity into durable pro-survival programs in glioma cells by initiating rapid Ca^2+^ influx events that act as the biochemical trigger for downstream kinase networks and transcriptional reprogramming [70]. Opening of Ca^2+^-permeable AMPA receptors (and voltage-dependent augmentation of NMDA receptor activity) during neuron→glioma synaptic transmission produces fast, high-amplitude intracellular Ca^2+^ transients that are both spatially and temporally encoded to activate calcium-sensitive effectors inside tumour cells [48]. These Ca^2+^ transients propagate within the malignant tissue as synchronized calcium waves through connected cells, converting focal synaptic events into a network-level signal that coordinates survival and repair responses across multicellular tumour ensembles [58]. At the molecular scale, the increase in intracellular Ca^2+^ quickly activates Ca^2+^/calmodulin-dependent kinases (CaMKs) and activates the phosphatase calcineurin; both of which additively modify immediate-early gene expression, post-translational modifications, and activity of transcription factors, which induce a cellular state primed for repair and ultimately proliferation [71]. Calcineurin-NFAT (Nuclear Factor of Activated T-cells) signaling, downstream of elevated Ca^2+^, forms a direct axis by which electrical activity alters transcriptional programs relevant to growth, migration and survival, thereby providing a mechanistic bridge from membrane depolarization to nuclear reprogramming [72]. Calcium-dependent mechanisms converge on the MAPK cascade: activity-induced inputs potentiate receptor tyrosine kinase signaling and RAF affect mitogen-activated extracellular signal-regulated kinase (MEK) and finally ERK phosphorylation cycles, which in turn upregulate cell-cycle drivers, pro-survival transcription factors, and DNA-repair facilitators [47]. Simultaneously, Ca^2+^ elevations support membrane recruitment and activation of PI3K, leading to AKT phosphorylation and downstream activation of mTOR pathway; this PI3K/AKT/mTOR axis provides an anabolic drive while additionally suppressing apoptosis and stimulating protein synthesis as necessary for recovery from genotoxic insults [73]. The crosstalk between MAPK and PI3K/AKT is synergistic rather than redundant: MAPK signaling reinforces transcriptional programs for proliferation, while PI3K/AKT maintains redox balance and repair competency, together shortening DNA-damage response latency and preserving clonogenic potential [74]. Sustained activation of these kinase networks is associated with epigenetic remodeling histone modifications and chromatin opening that locks in a neural-like malignant transcriptional state, making parts of the adaptive response semi-stable and enabling long-term tolerance to therapy [75]. A key metabolic effector that couples extracellular neurotransmitter dynamics to intracellular redox homeostasis is the cystine/glutamate antiporter SLC7A11 (xCT); its upregulation simultaneously exports glutamate (driving excitatory tone) and imports cystine to bolster glutathione synthesis, thereby shielding tumor cells from oxidative damage induced by radiotherapy and many chemotherapeutics [76]. SLC7A11-driven glutathione maintenance reduces lipid peroxidation and suppresses ferroptotic vulnerability, providing a concrete metabolic route by which neurotransmitter imbalance (glutamate excess) translates into pharmacologic resistance [77]. Beyond antioxidant defense, neuronal activity supplies substrates and signaling that rewire tumor metabolism—enhancing glutamine utilization, serine–glycine one-carbon flux, and NADPH production—that together support nucleotide synthesis and rapid repair of therapy-induced DNA damage [68]. The physical coupling of cells through tumor microtubes and adherens-type junctions permits intercellular propagation of electrical and calcium signals, creating a multicellular reservoir of repair capacity where undamaged regions can buffer and assist damaged cells, thereby blunting the efficacy of focal cytotoxic insults [78]. Intercellular calcium waves transmitted across these structural conduits are not passive spillover; they coordinate stress responses (e.g., recruitment of repair complexes, redistribution of metabolites) and may accelerate dissemination of resistance-conferring signaling molecules across the tumor field [79]. Crucially, Ca^2+^-dependent activation of kinase networks feeds directly into canonical DNA-damage response pathways: AKT and ERK signaling modulate homologous recombination and non-homologous end joining components, increase recruitment of repair effectors to double-strand breaks, and promote cell-cycle checkpoints favoring repair over apoptosis [74]. This mechanistic coupling explains why tumors embedded in active neuronal niches show faster recovery after genotoxic therapy and why blockade of neuronal inputs can sensitize models to radiation and alkylating agents. On the immunological front, neurotransmitter-driven metabolic and signaling changes create an immune-modulatory microenvironment: excess glutamate and activity-regulated trophic factors skew microglia/macrophage phenotypes toward tumor-supportive states and impair cytotoxic T-cell infiltration and function, further enabling resistant clones to persist [80]. Therapeutically, multiple non-overlapping intervention points emerge from this cascade: (i) blunt the initial Ca^2+^ surge (e.g., Ca^2+^-permeable AMPAR blockers or selective channel inhibitors), (ii) inhibit activity-regulated trophic signals (e.g., NLGN3 shedding inhibitors or TrkB antagonists), (iii) disrupt metabolic defenses (e.g., xCT inhibitors to lower glutathione and sensitize to ferroptosis), and (iv) target downstream kinase hubs (PI3K/AKT/mTOR or MEK/ERK inhibitors) in rational combinations. Preclinical evidence supports several of these approaches: blockade of Ca^2+^ permeable AMPAR subunits or pharmacologic inhibition of xCT reduces calcium transients, diminishes network propagation, and restores chemo-/radiosensitivity in xenograft and organoid models [81], as illustrated in Figure 3. However, each node has translational caveats: systemic AMPAR/TrkB inhibition risks neurocognitive side effects, xCT blockade can perturb peripheral redox balance, and PI3K/mTOR targeting must overcome toxicity and compensatory feedback loops; therefore, careful dosing windows, local delivery strategies, and biomarker-guided enrollment are essential [82]. From a trial-design perspective, early phase studies should incorporate “window-of-opportunity” biopsies and multimodal biomarker panels (tumor expression of SLC7A11, synaptic-gene signatures, functional connectivity mapping by fMRI/MEG, and CSF/plasma NLGN3 or BDNF levels) to link target engagement to mechanistic effects (e.g., reduced calcium wave propagation, decreased repair biomarkers) before assessing hard clinical endpoints [83]. Finally, an effective clinical strategy will likely require temporally sequenced combinations: attenuate neuron to tumor inputs during or immediately after cytotoxic exposure to prevent the early Ca^2+^-triggered repair program, then administer targeted inhibitors of PI3K/AKT or redox defenses to block residual survival signaling and metabolic adaptation, an approach that must be validated in carefully controlled translational studies. In sum, the Ca^2+^→MAPK/PI3K cascade sits at the heart of therapy resistance in neuron-engaged gliomas: it translates electrical activity into biochemical resilience, coordinates metabolic and epigenetic adaptations, and supports a multicellular networked resistance that demands multi-axis therapeutic strategies informed by circuit-level biomarkers [58].

### 5.4. Translational Strategies, Biomarkers, Delivery, Trial Design and a Roadmap for Clinical Implementation

Translating insights from cancer neuroscience into clinical benefit requires an integrated strategy that couples mechanistic biomarkers of neuron–tumour coupling with targeted interventions and delivery methods that achieve sufficient intratumoral exposure while limiting systemic neurotoxicity [84]. SLC7A11 (xCT) expression and function has emerged as both a mechanistic mediator and a candidate predictive biomarker for circuit-driven resistance, making quantification of SLC7A11 mRNA/protein and functional assays of cystine/glutamate flux a high-priority component of any biomarker panel [85]. Modulation of excitatory synaptic drive with clinically available AMPA-receptor antagonists is already feasible: pilot human studies testing perampanel for peritumoral hyperexcitability demonstrate the practicality of repurposing anti-seizure AMPAR blockers as adjuncts, and suggest signals worth pursuing in larger, biomarker-stratified trials [85]. Randomized translational trials nested around surgery, such as windowed perioperative studies that administer an anti-synaptic agent pre-resection and analyze treated tissue ex vivo, represent an efficient strategy to measure target engagement (e.g., reductions in synaptic gene expression, calcium transients, or NLGN3 release) before advancing to larger efficacy trials; examples of such perioperative designs are already being implemented (e.g., the PerSurge study) [86]. A structured development path for AMPAR-directed strategies should combine preclinical combination data (e.g., AMPAR antagonist + temozolomide) with phased dose-finding in humans that prioritizes neurocognitive endpoints as co-primary safety measures because of the central role of AMPAR signaling in normal brain function. For tumors with neurotrophic dependencies or NTRK fusions, TRK inhibitors provide an instructive precedent: orally bioavailable TRK inhibitors that penetrate the blood–brain barrier have shown clinical activity in selected CNS tumors and illustrate how molecularly targeted drugs can be repurposed or adapted to disrupt neuron-derived trophic signaling [87]. Nevertheless, not all neuron–tumor interactions are driven by a single targetable fusion; therefore, molecular profiling to identify NTRK fusions or upregulated neurotrophic receptor expression remains essential for rational patient selection in any targeted trial [88]. Optimizing intratumoral drug exposure is a recurring barrier—convection-enhanced delivery (CED) offers a viable route to deliver high local concentrations of agents that otherwise have poor brain penetration, and recent clinical and technical advances have improved both safety and distribution monitoring of CED infusions [89]. Demonstrations of CED with radiotherapeutic nanoliposomes and with other payloads in early-phase studies confirm that focal high-dose delivery can be achieved with tolerable safety profiles, supporting CED as a platform to test anti-synaptic biologics or metabolic inhibitors directly within the tumour bed [90]. Similarly, intratumoral infusion of cytotoxics and molecular inhibitors via CED (including liposomal formulations of agents like irinotecan) has been explored in phase I studies and provides a translational precedent and practical playbook for combining localized delivery with systemic cytotoxic therapy [91]. Because CED techniques and catheter strategies vary, compiling rigorous distribution imaging, pharmacokinetics, and tissue-biomarker endpoints into early studies is critical to establish dose–exposure–effect relationships for agents that target synaptic signaling or glutamate handling [92]. Ferroptosis-inducing strategies and nanoparticle-enabled redox modulation are attractive complements to circuit-directed therapies. By undermining tumor antioxidant defenses (for example, via xCT inhibition or by promoting lipid peroxidation), such approaches can exploit the metabolic dependencies imposed by neuron-driven glutathione synthesis [93]. Discovery and preclinical optimization of selective SLC7A11 inhibitors (and structure-guided lead molecules) have advanced rapidly and provide feasible drug candidates to combine with cytotoxic therapy or with AMPAR/TrkB modulation in rational combination trials [94]. Network-directed strategies that target physical tumor connectivity (for example, agents that disrupt tumor microtubules or gap-junction-like coupling) represent a complementary axis of attack. Preclinical evidence supports that weakening multicellular electrical/chemical conduits enhances focal therapy efficacy in vivo [58]. Because the malignant neurocircuitry exerts immunomodulatory effects, combining circuit-directed drugs with immunotherapy is an appealing but complex prospect; careful translational studies must elucidate whether modulating glutamate tone or trophic signaling augments or antagonizes anti-tumor immune function in situ [95]. Theoretically, non-pharmacologic neuromodulation modalities, e.g., focused ultrasound, noninvasive neuromodulation, or temporally targeted electrical stimulation could be applied to transiently suppress excitatory network activity that surrounds the tumor and subsequently mitigate calcium-triggered repair programs in the presence of cytotoxic exposure; early focused ultrasound studies and pilot safety data indicate that such a path is technically possible [96]. Pragmatically, trialists should adopt multi-stage designs that (a) run short “window-of-opportunity” arms to prove target modulation in resected tissue, (b) proceed to small randomized Phase II signal-seeking studies in biomarker-selected patients, and (c) only then attempt larger registration-focused trials with clinically meaningful endpoints [97]. Because these interventions carry potential central nervous system risks, trial protocols must pre-specify comprehensive neurocognitive batteries, high-resolution functional imaging (fMRI/MEG), and longitudinal CSF/plasma biomarker sampling to detect subtle neurotoxicity and to correlate circuit suppression with clinical benefit [98]. Regulatory and ethical considerations are non-trivial: first-in-human studies that attenuate neuronal signaling demand heightened safety monitoring, conservative dose escalation, and engagement with regulatory authorities early to define acceptable neurofunctional safety margins and stopping rules.

Finally, a realistic roadmap for clinical implementation couples (1) standardized biomarker panels (SLC7A11 expression, synaptic-gene signatures, CSF NLGN3/BDNF levels), (2) modality-specific delivery platforms (oral BBB-penetrant drugs, CED, intratumoral implants), and (3) staged trial designs prioritizing mechanistic proof before efficacy this integrated framework will maximize the chance that interventions which disrupt malignant neurocircuitry translate to measurable patient benefit [99].

## 6. Crosstalk Between Neurons, Glia, and Tumor Microenvironment

Previous studies have revealed multiple mechanisms underlying the crosstalk between astrocytes and tumor cells in brain metastases (BrM). For instance, metastatic cells release reelin, which attracts astrocytes that, in turn, secrete neuronal survival factors to promote tumor growth. In addition, astrocyte-derived extracellular vesicles transfer microRNAs into BrM cells, leading to the silencing of Pten and thereby driving tumor progression [100]. Another critical mechanism involves Stat3-activated astrocytes, which facilitate BrM establishment by suppressing CD8^+^ T cell responses and inducing the expansion of CD74^+^Iba1^+^ microglia/macrophage populations. Furthermore, tumor-infiltrating granulocytes stimulate pro-inflammatory astrocyte activation through lipocalin-2 signaling, creating a supportive microenvironment that enhances metastatic expansion [101]. 

### 6.1. Astrocyte-Mediated Immune Suppression in Brain Metastases

A critical functional difference exists between astrocyte subsets within the metastatic microenvironment. Tumor-associated astrocytes enhance Cdk5 expression in cancer cells, whereas naïve astrocytes exert a suppressive effect, highlighting their opposing roles in regulating metastatic progression [102]. This phenomenon intersects with mechanisms of immune evasion, as MHC-I molecules are indispensable for CD8^+^ cytotoxic T cells to detect and eliminate malignant cells [103]. During the early phases of tumor evolution, heterogeneous MHC-I expression is observed; however, under selective immune pressure, cells with higher MHC-I levels are eliminated, allowing for the survival and expansion of immune-evasive clones with diminished or absent expression. These variants, characterized by reduced antigenicity and increased aggressiveness, facilitate tumor progression and resistance to T cell–mediated surveillance [104]. In line with it has been reported that brain metastases (BrMs) evade immune recognition by downregulating MHC-I and APP expression, a mechanism supported by several studies in the field. Strikingly, pharmacological inhibition of Cdk5 with roscovitine (RSV) in vivo reversed this immunosuppressive program, restored MHC-I expression, and enhanced BrM responsiveness to immunotherapy, highlighting a promising therapeutic strategy to overcome immune resistance [103]. Moreover, astrocytes drive an increase in Cdk5 activity within BrM cancer cells, which in turn suppresses MHC-I expression and enables the tumor to avoid detection by CD8^+^ T cells. This immune evasion is mediated through Irf2bp1-dependent inhibition of the Stat1–importin α–Nlrc5 pathway, consistent with previous evidence highlighting the reciprocal communication between astrocytes and metastatic cells in shaping the tumor microenvironment [103].

### 6.2. Microglial Origins

#### 6.2.1. Characteristics of Microglial

Microglia represent a unique population of cells within the CNS, distinguished from neurons and other glial cells by their lineage and specialized functions. Unlike most CNS-resident cells that originate from the neuroectoderm, microglia arise from the yolk sac during embryonic development, establishing a hematopoietic lineage that defines their pivotal roles in brain immunity [105]. In addition to their role in development, microglial activity is controlled by metabolic processes, one of which is lipid metabolism. Maintaining homeostasis depends on appropriate lipid handling; however, dysregulated lipid metabolism impairs microglial function and has been linked to systemic diseases, including obesity, aging, and neurological disorders [106].

Functionally, microglia serve as the brain’s primary innate immune cells and act as first responders to injury, infection, and pathological insults. They account for approximately 5–12% of brain cells in mouse models, with proportions varying by brain region. Structurally, microglia display remarkable morphological diversity, ranging from compact amoeboid forms to elongated or radially branched states, each reflecting functional adaptation to local microenvironmental demands [107]. This structural neuronal plasticity is further underpinned by dynamic cytoskeletal remodeling. Transitions from homeostatic surveillance to reactive, pro-inflammatory phenotypes are orchestrated by microtubule network reorganization. In resting states, highly branched morphologies are maintained through stable microtubule arrays that facilitate constant environmental monitoring [108]. Upon activation, however, microglia undergo a dramatic shift driven by Cdk1 activity independent of cell division, involving centrosome activation, inhibition of Golgi-based microtubule nucleation, suppression of Stathmin 1, and stabilization of microtubules through MAP4. These cytoskeletal adaptations are essential for the adoption of amoeboid morphology, cytokine trafficking, and secretion, thereby linking microtubule dynamics to inflammatory signaling and neuroimmune responses [109].

#### 6.2.2. Microglia and Tumor-Associated Macrophages in Glioblastoma

Microglia are central contributors to the tumor microenvironment in GBM, where they are frequently co-opted into tumor-associated macrophages (TAMs) that facilitate tumor progression. Multiple therapeutic strategies have been explored to target these TAMs, including depletion approaches such as CD11b-HSVTK, clodronate, and Ccl2 reduction, which aimed to eliminate tumor-supporting populations [110]. Although such interventions reduced tumor size in certain instances, paradoxical tumor growth of up to 33% was observed, particularly when applied before tumor establishment. An alternative strategy involves repolarizing immunosuppressive, pro-tumor TAMs (M2) into anti-tumor phenotypes (M1). Therapeutic modalities in this category include CSF-1R inhibition although tumors can adapt through PI3K/IGF-1 signaling along with STAT3 blockade, nanoparticle-mediated delivery of IRF-5/IKKβ mRNA, and IL-33 targeting [111]. While these approaches show promise, they remain constrained by compensatory pathways. Another avenue capitalizes on enhancing microglial phagocytosis, as GBM cells evade immune clearance by exploiting the “don’t eat me” signal CD47. Anti-CD47 antibodies restore microglial phagocytic activity while limiting inflammation, and combination regimens integrating Anti-CD47 with Temozolomide and Anti-PD1 therapy have demonstrated superior anti-tumor efficacy [112].

#### 6.2.3. Oligodendrocyte

The oligodendrocyte lineage is composed of oligodendrocyte progenitor cells (OPCs), also known as NG2 glia, and terminally differentiated mature oligodendrocytes (OLs). The designation “NG2 glia” is frequently applied to OPCs because they express the NG2 proteoglycan (CSPG4). Within the CNS, OPCs are considered the fourth major glial cell type, distinct from astrocytes, microglia, and mature oligodendrocytes. Since NG2 is also expressed by pericytes, the term “NG2 glia” provides a more precise description of this progenitor population [113]. Unlike existing oligodendrocytes, new myelin is typically generated through the differentiation of OPCs. A unique feature of these progenitors is that they are not restricted to specific niches but are evenly distributed throughout the CNS, accounting for approximately 5% of adult mouse CNS cells. Each OPC extends a branched network of processes, occupying its own distinct territory and expanding into unoccupied regions via migration, growth, or proliferation, effectively “tiling” the CNS [114]. Beyond their role in generating myelinating oligodendrocytes, OPCs exhibit distinctive physiological properties that tightly integrate them into neural circuits. They express a range of neurotransmitter receptors and voltage-gated ion channels, enabling them to sense neuronal activity with remarkable spatial and temporal precision, and they can form postsynaptic contact with neurons. These characteristics indicate that OPCs participate in neural network dynamics in ways that extend beyond their classical role in myelination [115].

### 6.3. Oligodendrocytes & Lactylation

#### 6.3.1. Histone Lactylation in Oligodendrocytes

Oligodendrocytes, the myelinating cells of the CNS, depend on high metabolic activity to sustain their functions. Yet, in pathological conditions such as multiple sclerosis (MS), gliomas, and other neurodegenerative disorders, they often face metabolic stress associated with elevated lactate levels. Traditionally recognized as a key energy metabolite in the astrocyte–neuron lactate shuttle, lactate has more recently been identified as an epigenetic regulator through histone lactylation. In gliomas, which frequently arise from oligodendroglial lineage, histone lactylation remodels chromatin architecture and reprograms oligodendrocyte lineage cells, thereby facilitating malignant transformation [116]. Within the tumor microenvironment, increased lactate concentrations further drive histone lactylation, resulting in global transcriptional reprogramming. In oligodendrocyte progenitor cells (OPCs), this epigenetic modification activates oncogenic signaling pathways such as NF-κB and PDGFRβ, ultimately contributing to gliomagenesis and malignant progression [117].

#### 6.3.2. Neuron–Tumor Crosstalk

Neuron–tumor crosstalk represents a dynamic and reciprocal interaction between glioma cells and surrounding neurons that critically contributes to tumor progression. A key element of this process is the activity-dependent release of NLGN3, which is shed by both neurons and OPCs through ADAM10-mediated cleavage. The released NLGN3 activates PI3K–mTOR oncogenic signaling within glioma cells, thereby enhancing their proliferative capacity [118]. In parallel, glioma cells establish direct synaptic contact with neurons, forming functional glutamatergic synapses. Through these neuron-glioma connections, excitatory signals are transmitted via AMPA-type glutamate receptors, generating excitatory postsynaptic currents (EPSCs) in tumor cells. This synaptic coupling promotes tumor progression by driving electrical activity that stimulates further proliferation [81]. Moreover, glioma cells amplify network hyperexcitability by releasing glutamate primarily through the overactive cystine glutamate antiporter resulting in elevated extracellular glutamate concentrations. This not only reinforces excitatory signaling but also disrupts inhibitory pathways, including those mediated by GABAergic interneurons, thereby creating a permissive environment for tumor expansion.

#### 6.3.3. Therapeutic Strategies Targeting Neuron–Glioma Communication Pathways

Disrupting the harmful neuron–glioma crosstalk provides a promising therapeutic strategy. One approach targets the metalloprotease ADAM10 to inhibit the activity-dependent release of soluble NLGN3, a secreted synaptic protein that drives glioma proliferation. Preclinical studies using patient-derived high-grade glioma xenograft models demonstrated that ADAM10 inhibition significantly reduces tumor growth by preventing NLGN3 shedding. Equally compelling is the blockade of AMPA-type glutamate receptor signaling, which mediates excitatory currents from neuron–glioma synapses. In this context, the AMPAR antagonist perampanel suppressed glioma proliferation in vivo, while competitive inhibitors such as NBQX reduced glioma growth only under neuron co-culture conditions [55].

The tumor microenvironment involves not only neurons but also complex interactions with glial cells, particularly astrocytes. Astrocytes are the most abundant type of glial cells and were identified alongside neurons, although their role was historically regarded as merely structural support for brain integrity. Advances in technology have since revealed astrocytes to be critical regulators of brain physiology, behavioral responses, and pathological processes [119]. Morphologically, astrocytes are highly intricate, star-shaped cells that establish extensive connections throughout the CNS. They are multifunctional, maintaining ion and neurotransmitter balance, facilitating synapse formation and elimination, modulating synaptic activity, and contributing to neurovascular coupling and blood–brain barrier integrity [120]. In neurological and psychiatric disorders, astrocytes play central roles; and although traditionally considered a homogeneous population in contrast to the diversity of neurons, emerging gene expression analyses and functional studies in both health and malignancy have revealed regional and functional heterogeneity among astrocytes [121].This recognition of astrocyte diversity, combined with their intricate morphology, suggests that they are capable of fulfilling highly specialized functions across distinct CNS regions. Nonetheless, a comprehensive molecular characterization of astrocyte diversity, similarities, and morphological traits across the entire CNS remains lacking in any species [122].

### 6.4. The Glutamate–Glutamine Cycle

#### 6.4.1. The Glutamate–Glutamine Cycle: A Core Mechanism of Excitatory–Inhibitory Balance in the CNS

In the central nervous system, Glu functions as the principal excitatory neurotransmitter, whereas GABA serves as its major inhibitory counterpart. The glutamate–glutamine cycle (GGC) represents a critical pathway interlinking the metabolism and recycling of these two neurotransmitters, thereby maintaining synaptic homeostasis and mediating neuron–astrocyte interactions. In this cycle, glutamate released into the synaptic cleft is rapidly taken up by astrocytes through excitatory amino acid transporters, predominantly GLAST/EAAT1 and GLT1/EAAT2 [123]. Within astrocytes, glutamate is converted into glutamine by the enzyme glutamine synthetase (GS), which is specifically expressed in astrocytic cells. The glutamine produced is subsequently exported and delivered back to neurons via the sodium-coupled neutral amino acid transporters SNAT1 and SNAT2. Once inside neurons, phosphate-activated glutaminase (PAG) catalyzes the reconversion of glutamine into glutamate, thereby completing the GGC and sustaining excitatory neurotransmission. The regenerated glutamate can either act directly as a neurotransmitter or serve as a precursor for GABA synthesis through the action of GAD in inhibitory neurons. This tightly regulated process ensures efficient clearance of synaptic glutamate to prevent excitotoxicity while simultaneously maintaining continuous nitrogen exchange and metabolic integration between neuronal and glial compartments [124].

#### 6.4.2. Pathological Disruptions and Therapeutic Potential of SCFAs

**Figure 4:**
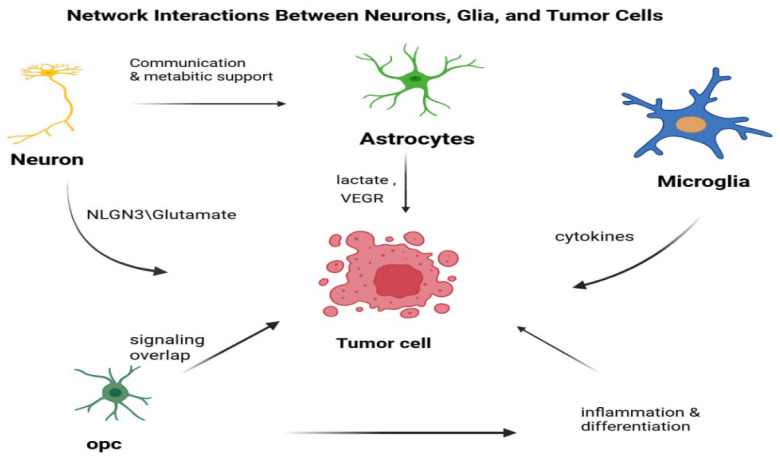
Cellular crosstalk within the neural microenvironment: neurons and different glial cell types (astrocytes, microglia, and other support cells) interact dynamically with tumor cells, influencing tumor growth, immune responses, and neural function. Created by Biorender, (Biorender.com, accessed on 3 December 2025).

Many types of neurological diseases have been associated with GGC disruptions, both in terms of their development and their progression. Studies conducted in transgenic mice have shown that short-chain fatty acids (SCFAs), which are produced by microbes fermenting dietary fibers, have several benefits. These include improving learning and memory performance, reducing amyloid-β accumulation and abnormal Tau phosphorylation, and enhancing glial-neuronal metabolic interactions. These findings suggest that SCFAs may contribute to restoring GGC function and supporting overall brain metabolism [125]. At the molecular level, the efficient operation of the GGC relies on complex transport machinery. Excitatory amino acid transporters (EAATs) are expressed in both neurons and astrocytes, with GLAST/EAAT1 and GLT1/EAAT2 representing the predominant astrocytic isoforms responsible for tightly regulating extracellular glutamate levels and thereby protecting neurons from excitotoxicity [126]. In parallel, multiple glutamine transport systems sustain the bidirectional exchange between neurons and astrocytes. System A (SNAT1, SNAT2), expressed in both cell types, mediates glutamine uptake; System N (SNAT3, SNAT5), largely confined to astrocytes, is critical for exporting glutamine to neurons; System ASC (ASCT1, ASCT2) facilitates bidirectional exchange of glutamine with smaller amino acids such as serine, alanine, and cysteine; and System L (LAT1, LAT2) regulates the transport of larger neutral amino acids, including leucine and histidine, while also contributing modestly to glutamine transfer. Also, previous studies have revealed multiple mechanisms underlying the crosstalk between astrocytes and tumor cells in BrM, as illustrated in Figure 4. Collectively, these transport pathways maintain the equilibrium between excitatory and inhibitory neurotransmission and ensure effective metabolic coupling between astrocytes and neurons [127].

## 7. Therapeutic Opportunities Targeting Neuronal Plasticity

### 7.1. Pharmacological Approaches

Glutamate inhibitors, AMPA/NMDA receptor antagonists, and GABA agonists represent complementary strategies for targeting glioma progression and restoring excitatory–inhibitory balance in the central nervous system. Sulfasalazine, for instance, acts by inhibiting the cystine–glutamate antiporter (system Xc^−^, mediated by xCT/SLC7A11), thereby reducing pathological glutamate release from glioma cells. This not only suppresses glioma proliferation and invasion but also mitigates tumor-associated excitotoxicity and seizures, with recent in vivo studies showing that sulfasalazine administration significantly diminishes glioblastoma invasion by lowering extracellular glutamate concentrations [62]. In parallel, perampanel a selective, noncompetitive AMPA receptor antagonist originally approved as an antiepileptic drug has been shown to suppress neuronal hyperexcitability and reduce seizure activity in clinical epilepsy settings and in vivo experimental models Beyond seizure control, preclinical studies highlight its ability to minimize hippocampal hyperexcitability in amyloid-β models and to inhibit glioblastoma proliferation and glutamate release in vitro, while the results from the phase II PerSurge trial provide preliminary clinical evidence supporting a potential role in restricting glioblastoma invasion and proliferation alongside a favorable safety and tolerability profile [128]. Complementing these approaches, strategies aimed at enhancing GABAergic activity offer a means to restore inhibitory control within disrupted neuronal circuits. Activation of GABA_A and GABA_B receptors through agonists or positive allosteric modulators helps reestablish excitatory–inhibitory equilibrium, with neuroactive steroids such as allopregnanolone providing anxiolytic, anticonvulsant, and neuroprotective effects by potentiating GABA_A receptor activity [129]. Importantly, dysregulation of GABA receptor trafficking, expression, or degradation has been implicated in neurological pathologies, further underscoring the therapeutic potential of interventions that restore GABAergic receptor function to stabilize neural networks.

### 7.2. Computational & AI Approaches

#### 7.2.1. Modeling Tumor–Neuron Networks

Computational approaches have become indispensable for unraveling the dynamics of tumor–neuron interactions and the adaptive behavior of glioblastoma within the brain microenvironment. Techniques such as rabies-based retrograde tracing have mapped tumor–neuron connectivity across the brain, showing that tumors rapidly integrate into neural circuits to drive invasion and spread. Therapeutic strategies targeting this crosstalk are gaining momentum; for example, preclinical modeling and experimental studies suggest that combining radiotherapy with perampanel can disrupt tumor-associated neuronal networks and enhance treatment efficacy. In contrast, selective silencing of tumor-connected neurons can entirely block tumor progression [130]. At a broader systems level, whole-brain modeling coupled with functional connectivity data has revealed that tumors reshape brain dynamics by amplifying inter-regional neuronal communication, thereby contributing to abnormal activity patterns. In parallel, mathematical models describing tumor evolution as dynamic networks of cellular states indicate that gliomas rely on flexible hierarchical architectures, with transition rates that vary among patients and adapt dynamically under therapeutic pressure [131]. Extending beyond neuronal network integration, computational models of exploratory adaptation further emphasize how glioblastoma cells exploit stochastic genetic mutations to adjust to diverse microenvironmental constraints. This neuronal plasticity provides a mechanistic explanation for their survival and persistence across distinct brain regions, underscoring the necessity of incorporating both evolutionary dynamics and microenvironmental context into predictive modeling frameworks.

#### 7.2.2. Personalized Therapy Prediction: Integrating AI, Neuroinformatics & Omics

**Figure 5:**
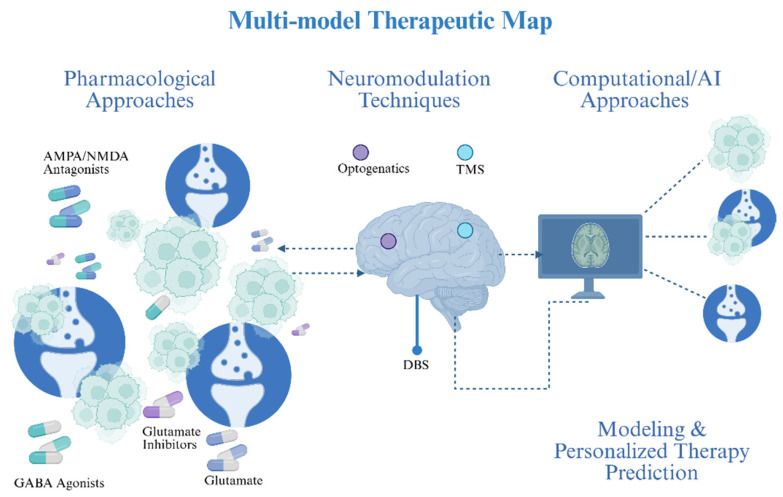
Multi-model Therapeutic Map strategies targeting tumor-neuron interactions. Created by Biorender, (Biorender.com, accessed on 3 December 2025).

**Figure 6:**
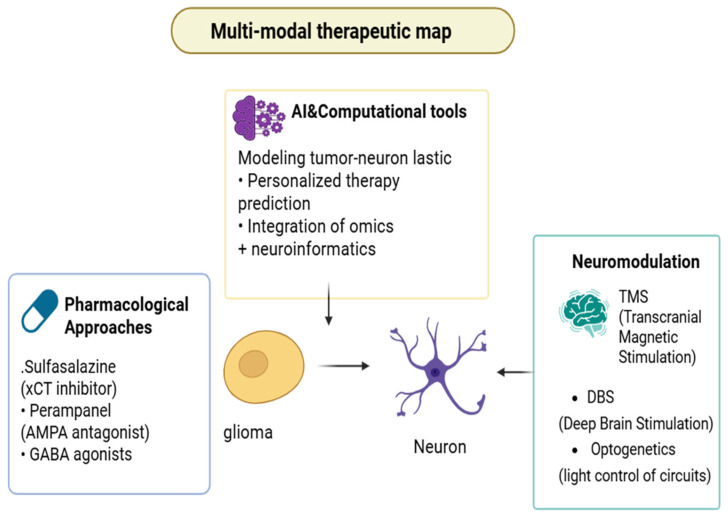
Multi-modal therapeutic approach for tumor-neuron interactions. Therapeutic map that integrates three main strategies: pharmacological approaches, AI and computational tools, and neuromodulation. Pharmacological interventions, such as Sulfasalazine, Perampanel, and GABA agonists, target tumor-neuron interactions. AI tools assist in modeling these interactions for personalized therapy predictions and integrating omics and neuroinformatics data. Additionally, neuromodulation techniques like TMS (Transcranial Magnetic Stimulation), DBS (Deep Brain Stimulation), and optogenetics provide alternative methods for controlling neural circuits. Created by Biorender, (Biorender.com, accessed on 3 December 2025).

Artificial intelligence (AI) has become a cornerstone of personalized medicine, offering the ability to integrate diverse datasets, including neuroimaging radiomics, multi-omics information, and their interactions to predict individual treatment responses with high precision. Applications of AI already span tumor diagnosis through imaging, optimization of surgical and radiotherapy planning, and the integration of heterogeneous data modalities to tailor therapeutic strategies while also enabling accurate prediction of survival outcomes and recurrence [132]. Within specialized analytical modeling, machine learning algorithms have proven particularly effective in generating individualized survival estimates, and practical web-based tools now allow clinicians to apply these models for precise patient-specific risk assessments [17]. Beyond clinical prediction, the integration of multi-layered omics data, including genomics, transcriptomics, proteomics, and epigenomics, offers a deeper understanding of intratumor heterogeneity and supports the discovery of novel diagnostics. The integrated conceptual model as illustrated in Figure 5 and Figure 6 is aligned with the therapeutic landscape summarized in Table 1, providing both a visual and tabulated representation of current strategies modulating tumor–neuron network dynamics.

## 8. Clinical and Pre-Clinical Studies of Neural Plasticity in Brain Tumor Progression

Brain tumors such as gliomas dynamically interact with neural plasticity, where the brain’s capacity to recognize circuits can mitigate deficits while promoting tumor progression. Preclinical models, including patient-derived xenografts and optogenetic manipulations in mice, demonstrate how neural activity fosters glioma growth via glutamatergic and GABAergic synapses [138]. For instance, Brain-Derived Neurotrophic factor (BDNF) enhances AMPA receptor trafficking, increasing depolarization and proliferation in pediatric gliomas by ~10% (*p* = 0.016), with Tropomyosin-related kinase B receptor (TrkB) knockout extending survival [48]. mTOR inhibition reverses tumor-induced neural hyperexcitability, reducing seizures and cognitive deficits, where TrkB inhibition extending survival (*p* < 0.05). These findings underscore plasticity’s role in tumor-neuron crosstalk [139]. For clinical implications, In patients, neuroimaging functional Magnetic Resonance Imaging (fMRI), Diffusion Tensor Imaging (DTi) reveals reorganization to contralesional hemispheres in low-grade gliomas, preserving language function during surgery [140]. For instance, in a cohort of 13 glioma patients, 92% exhibited plasticity, correlated with tumor proximity (<2 cm to motor areas increasing contralesions recruitment, *p* = 0.04). Multistage surgeries maintain Karnofsky scores >80% for over 12 years in IDH-mutant Isocitrate Dehydrogenase-mutant Low-Grade Gliomas (LGGs) [141]. High-neural epigenetic signatures correlate with reduced survival and increased synaptic integration, suggesting maximized resection for better outcomes [142]. However, rapid tumor growth in high-grade cases may outpace adaptive plasticity [143]. Emerging evidence from small-scale trials shows neuromodulation techniques, such as repetitive Transcranial Magnetic Stimulation (rTMS) and tDCS, may enhance pre-surgical plasticity to improve resection safety, but the results are preliminary and depend on targeting accuracy [144].

## 9. Challenges and Limitations in Targeting Neuronal Plasticity in Brain Tumors

Although more individuals are starting to understand that neuronal plasticity is a major cause of brain tumor growth, network integration, and therapy resistance, there are still a lot of problems and limitations that make it hard for us to fully understand how plasticity-directed therapies work and how to use them in the clinic [48]. A significant limitation emerges from the biological intricacy and variability in gliomas. Glioblastoma, the typical aggressive brain tumor, demonstrates significant inter- and intra-tumoral variability in genetic, epigenetic, and phenotypic characteristics, facilitating fast adaptability to various treatment approaches [132]. The ability of cells to alter their morphology enables glioma cells to transition between several molecular states and stem-like phenotypes. Also, this makes targeted treatments less effective and makes it more likely that the disease will come back and not respond to treatment after the first one. This type of plasticity makes it harder to find universal therapeutic targets and makes treatments with only one drug less effective [145]. The absence of reliable predictive biomarkers is closely associated with tumor heterogeneity. Biomarkers that classify patients based on neuron–tumor connectivity or plasticity signatures remain inadequately validated. Molecular markers like MGMT promoter methylation can tell us how well a tumor will respond to temozolomide, but they do not show how neuronal activity affects how the tumor behaves [146]. The lack of strong, reproducible biomarkers makes it hard to choose patients who are most likely to benefit from circuit-directed therapies or to keep track of how well they are responding to treatment [132]. From a clinical standpoint, the risk of neurotoxicity constitutes a considerable obstacle to therapies aimed at neuronal signaling. Many neural receptors and pathways that are involved in tumor growth, such as AMPA and NMDA glutamate receptors, are also necessary for normal brain functions such as memory and learning [147]. If neural specificity is not achieved, the antagonism of these pathways may impede essential brain functions. One of the biggest problems with the drugs and brain-altering methods we have now is that they cannot kill tumors and keep neurons alive at the same time [148]. Finally, Current strategies for assessing and modulating neural plasticity remain limited. Indirect techniques such as MRI imaging and magnetoencephalography provide insights into large-scale network activity; however, their restricted spatial and temporal resolution precludes a comprehensive characterization of dynamic neuron–tumor interactions at the cellular and synaptic levels. Moreover, emerging interventional modalities, including deep brain stimulation and optogenetics, require substantial methodological refinement and rigorous translational validation before they can be safely and effectively integrated into routine clinical practice [147].

## 10. Future Perspectives

Cancer neuroscience has recently emerged as a distinct interdisciplinary discipline, merging perspectives from neurobiology, oncology, immunology, and computational sciences to unravel the influence of neural processes on tumor development and progression [149]. With advances in network neuroscience and connect omics, researchers can now quantitatively model brain network organization and its pathological alterations, tools that are increasingly being applied to characterize tumor-driven circuit rewiring and inform therapeutic strategies [150]. The integration of connect omics, neuroinformatics, and multi-omics presents a major paradigm shift. Functional and structural connectome mapping using modalities such as resting-state fMRI and diffusion MRI provides insights into how tumors perturb neural networks and facilitates individualized neurosurgical planning [151]. At the same time, multi-omics approaches spanning genomics, epigenomics, transcriptomics, and proteomics, when combined through systems bioinformatics and machine learning, enable deeper tumor characterization and identification of novel biomarkers [152]. Furthermore, Recognition is growing that therapies designed to target the neuron–tumor interface could be personalized by leveraging such integrative data. Patient-specific connect omics alterations may help guide tailored interventions whether through neuromodulation, pharmacological targeting, or AI-assisted predictive modeling that aim to disrupt maladaptive synaptic interactions [153]. Likewise, comprehensive multi-omics profiling can stratify patients for precision therapies aimed at neuronal plasticity-associated signaling pathways [154]. Nonetheless, translating these approaches into clinical practice remains challenging. Obtaining high-resolution multimodal connectomes and omics datasets in real-world settings is constrained by resource demands, technical variability, and lack of standardized protocols [155]. Furthermore, converting these complex datasets into clinically actionable models requires advanced computational infrastructure and interpretable algorithms, a task complicated by the small sample sizes typical of brain tumor research Addressing these limitations will necessitate cross-disciplinary collaborations to build shared multimodal repositories, develop transparent AI pipelines, and rigorously validate neuron–tumor–targeted strategies in preclinical and clinical trials 

## 11. Conclusions

Neuronal plasticity is a core feature of the brain, underpinning its ability to adapt, reorganize, and preserve function across the lifespan. In brain tumors, however, this same property is hijacked to serve malignant ends. Gliomas and other primary brain cancers co-opt processes such as synaptic remodeling, neurotransmitter release, and activity-dependent signaling to fuel their growth, invasion, and resistance to therapy. The identification of functional neuron–glioma synapses and excitatory glutamatergic circuits has redefined these tumors, shifting the paradigm from viewing them solely as genetic or molecular disorders to understanding them as complex “neuro-oncological network diseases.” This shift exposes the shortcomings of traditional approaches that focus exclusively on tumor genomics without considering the broader neuronal milieu. Cancer neuroscience has thus brought the neuron–tumor interface into focus as a novel therapeutic target. With the advent of connect omics, neuroinformatics, and multi-omics platforms, researchers can now chart tumor-associated neural circuits with remarkable detail. These insights pave the way for individualized treatment strategies that not only attack the cancer cells themselves but also disrupt the pathological neural networks that sustain them. Approaches including neurotransmitter modulation, neuromodulation technologies, and artificial intelligence-based predictive tools hold potential to recalibrate aberrant neuronal plasticity while safeguarding essential brain function. Nonetheless, important challenges remain. The intricate nature of the brain microenvironment, the possibility of unintended harm to healthy neural networks, and the difficulty of monitoring plasticity over time all present barriers to clinical application. Addressing these issues will demand close collaboration among neuroscientists, oncologists, engineers, and computational biologists, alongside rigorously designed clinical trials that combine functional biomarkers with meaningful patient outcomes. By re-envisioning neuronal plasticity as both a contributor to malignancy and a point of therapeutic intervention, cancer neuroscience provides a powerful framework for advancing precision medicine. Leveraging this dual role of neuronal plasticity may ultimately enable the development of more effective, durable, and personalized strategies against brain tumors.

## Figures and Tables

**Table 1:** Emerging therapeutic strategies targeting neuron–glioma network interactions. The table outlines primary molecular or circuit-level targets, proposed mechanisms of action, and the status of preclinical or clinical evidence supporting each approach.

Therapeutic Strategy	Primary Target	Proposed Mechanism	Experimental/Clinical Evidence	Reference
Sulfasalazine	xCT cystine–glutamate exchanger	Suppresses glutamate release	Preclinical evidence + small pilot clinical studies	[133]
Perampanel	AMPA-type glutamate receptors	Reduces neuronal hyperactivity & glioma proliferation	Preclinical studies; ongoing clinical trials	[134,135]
GABAergic Agonists	GABA-A receptor complexes	Restores inhibitory signaling	Preclinical models only	[62]
Optogenetic Modulation	Neuron–tumor synaptic contacts	Direct control of excitability	Animal model validation	[136]
TMS/DBS	Cortical and subcortical circuits	Neuromodulation of activity	Pilot/early-stage studies	[137]

## Data Availability

No new data were created or analyzed in this study.

## Abbreviations

AI	Artificial Intelligence
AMPA	α-amino-3-hydroxy-5-methyl-4-isoxazolepropionic acid
CNS	Central Nervous System
DBS	Deep Brain Stimulation
EGFR	Epidermal Growth Factor Receptor
GBM	Glioblastoma Multiforme
IDH	Isocitrate Dehydrogenase
LTP	Long-Term Potentiation
MAPK	Mitogen-Activated Protein Kinase
NMDA	N-methyl-D-aspartate (receptor)
PDX	Patient-Derived Xenograft
PI3K/AKT	Phosphoinositide 3-Kinase/Protein Kinase B Pathway
TMS	Transcranial Magnetic Stimulation
WHO	World Health Organization
AKT	Protein Kinase B
AMPAR	AMPA Receptor
BDNF	Brain-Derived Neurotrophic Factor
BBB	Blood–Brain Barrier
CED	Convection-Enhanced Delivery
CIC	Capicua Transcriptional Repressor
CSF	Cerebrospinal Fluid
DNA	Deoxyribonucleic Acid
EAAT	Excitatory Amino Acid Transporter
ERK	Extracellular Signal-Regulated Kinase
GABA	Gamma-Aminobutyric Acid
GSC	Glioma Stem-like Cell
GSCs	Glioma Stem-like Cells
IJMCM	International Journal of Molecular and Cellular Medicine
MEK	Mitogen-activated extracellular signal-regulated kinase
MEG	Magnetoencephalography
NADPH	Nicotinamide Adenine Dinucleotide Phosphate (reduced form)
NFAT	Nuclear Factor of Activated T-cells
NKCC1	Sodium-Potassium-Chloride Cotransporter 1
RSV	Roscovitine
NLGN3	Neuroligin-3
NTRK	Neurotrophic Receptor Tyrosine Kinase
PI3K	Phosphoinositide 3-Kinase
mTOR	Mechanistic Target of Rapamycin
TrkB	Tropomyosin receptor kinase B
SLC7A11/xCT	Cystine/Glutamate Antiporter
CaMK	Calcium/Calmodulin-Dependent Protein Kinase
fMRI	Functional Magnetic Resonance Imaging
BrM	Brain Metastases
CD8^+^ T cells	Cytotoxic T lymphocytes (CD8-positive T cells)
MHC-I	Major Histocompatibility Complex class I
Cdk5	Cyclin-dependent kinase 5
TAMs	Tumor-associated macrophages
OPCs	Oligodendrocyte progenitor cells
NG2 glia	Oligodendrocyte progenitor cells expressing the NG2 proteoglycan
OLs	Oligodendrocytes
SCFAs	Short-chain fatty acids
xCT	Cystine–glutamate exchanger
GABA-A receptors	Gamma-aminobutyric acid type A receptors
MAP4	Microtubule-associated protein 4
NF-κB	Nuclear factor kappa-light-chain-enhancer of activated B cells
PDGFRβ	Platelet-derived growth factor receptor beta
IRF-5	Interferon regulatory factor 5
IKKβ	IκB kinase beta
CD47	Cluster of differentiation 47
MGMT	O-6-methylguanine-DNA methyltransferase
HGG	High-Grade Gliomas
ADAM10	A Disintegrin and Metalloprotease 10
DMG	Diffuse Midline Gliomas
GABAA	Gamma-Aminobutyric Acid Type A Receptor
TMs	Tumor Microtubes
PVN	Perivascular Niche
NOTCH1	Notch Receptor 1
LGNTs	Low-Grade Neuroepithelial Tumors
IUE	In Utero Electroporation
TSP1	Thrombospondin-1
KCC2	Potassium-Chloride Cotransporter 2
CD8+ T-cells	Cluster of Differentiation 8-positive T-cells
LTD	Long-term depression
NMDAR	N-methyl-D-aspartate receptor
NMDARs	N-methyl-D-aspartate receptors
NMDAR-LTD	NMDA receptor-dependent long-term depression
mGluR-LTD	Metabotropic glutamate receptor-dependent long-term depression
EPSPs	Excitatory postsynaptic potentials
CA3	Cornu ammonis area 3 of the hippocampus
IHP	Hyperpolarization-activated Current/Ih
G-protein-coupled	Guanine nucleotide-binding protein-coupled
GIRK	G-protein-coupled inwardly rectifying potassium channels
cAMP	cyclic adenosine monophosphate
CREB	cAMP response element-binding protein
H-channels	hyperpolarization-activated cyclic nucleotide-gated (HCN) channels
tDCS	transcranial direct current stimulation
Glu	Glutamate
NT	Neurotransmitter
Glu MRS	Glutamate Magnetic Resonance Spectroscopy
ECS	Extracellular space
(GAD)-67	Glutamic acid decarboxylase-67
TrkB	Tropomyosin-related kinase B receptor
DTi	Diffusion Tensor Imaging
rTMS	Transcranial Magnetic Stimulation
